# Single-cell RNA sequencing of mouse brain and lung vascular and vessel-associated cell types

**DOI:** 10.1038/sdata.2018.160

**Published:** 2018-08-21

**Authors:** Liqun He, Michael Vanlandewijck, Maarja Andaloussi Mäe, Johanna Andrae, Koji Ando, Francesca Del Gaudio, Khayrun Nahar, Thibaud Lebouvier, Bàrbara Laviña, Leonor Gouveia, Ying Sun, Elisabeth Raschperger, Åsa Segerstolpe, Jianping Liu, Sonja Gustafsson, Markus Räsänen, Yvette Zarb, Naoki Mochizuki, Annika Keller, Urban Lendahl, Christer Betsholtz

**Affiliations:** 1 Department of Neurosurgery, Tianjin Medical University General Hospital, Tianjin Neurological Institute, Key Laboratory of Post-Neuroinjury Neuro-Repair and Regeneration in Central Nervous System, Ministry of Education and Tianjin City, Tianjin 300052, China.; 2 Integrated Cardio Metabolic Centre, Department of Medicine Huddinge, Karolinska Institutet, Blickagången 6, SE-141 57 Huddinge, Sweden.; 3 Department of Immunology, Genetics and Pathology, Rudbeck Laboratory, Uppsala University, Dag Hammarskjölds väg 20, SE-751 85 Uppsala, Sweden.; 4 Department of Cell and Molecular Biology, Karolinska Institutet, Von Eulers väg 3, SE-171 77 Stockholm, Sweden.; 5 Inserm U1171, University of Lille, CHU, Memory Center, Distalz, F-59000 Lille, France.; 6 Department of Bioinformatics, Zhongyuan Union Genetic Technology Co., Ltd., No.45, the 9th East Road, Tianjin airport economic area, Tianjin 300304, China.; 7 Wihuri Research Institute and Translational Cancer Biology Program, Biomedicum Helsinki, University of Helsinki, Haartmaninkatu 8, P.O. Box 63, FI-00014 Helsinki, Finland.; 8 Division of Neurosurgery, Zürich University Hospital, Zürich University, Zürich, CH-8091, Switzerland.; 9 Department of Cell Biology, National Cerebral and Cardiovascular Center Research Institute, 5-7-1 Fujishirodai, Suita, Osaka 565-8565, Japan.; 10 AMED-CREST, National Cerebral and Cardiovascular Center, 5-7-1 Fujishirodai, Suita, Osaka 565-8565, Japan.

**Keywords:** Mouse, RNA sequencing, Molecular neuroscience, Gene expression analysis

## Abstract

Vascular diseases are major causes of death, yet our understanding of the cellular constituents of blood vessels, including how differences in their gene expression profiles create diversity in vascular structure and function, is limited. In this paper, we describe a single-cell RNA sequencing (scRNA-seq) dataset that defines vascular and vessel-associated cell types and subtypes in mouse brain and lung. The dataset contains 3,436 single cell transcriptomes from mouse brain, which formed 15 distinct clusters corresponding to cell (sub)types, and another 1,504 single cell transcriptomes from mouse lung, which formed 17 cell clusters. In order to allow user-friendly access to our data, we constructed a searchable database (http://betsholtzlab.org/VascularSingleCells/database.html). Our dataset constitutes a comprehensive molecular atlas of vascular and vessel-associated cell types in the mouse brain and lung, and as such provides a strong foundation for future studies of vascular development and diseases.

## Background & Summary

**Figure 1:**
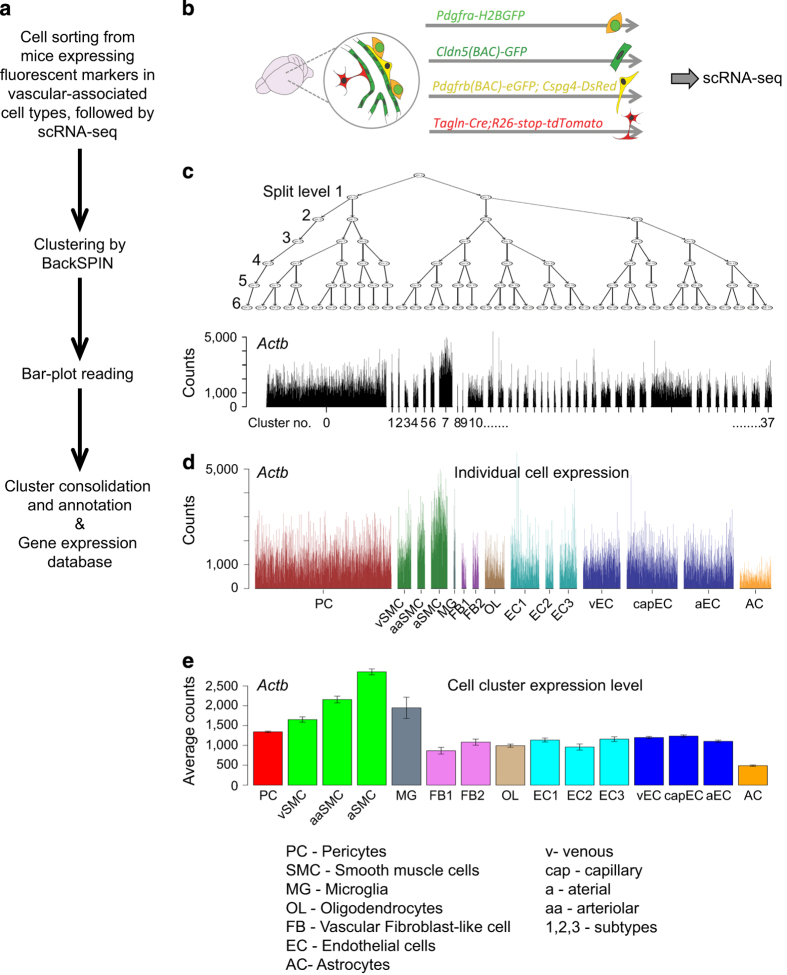
Work flow. (**a**) Flowchart of the procedure of generating the single cell dataset. (**b**) Whole adult mouse brains from the indicated mouse reporter lines were mechanically and enzymatically digested, and single cells were isolated by FACS, cDNA libraries prepared and sequenced. (**c**) Single cell transcriptomes were clustered by BackSPIN. The black bar-plot shows *Actb* expression (sequence counts) in the 38 clusters (0–37) generated at split-level 6: these clusters were given a preliminary cell class assignment (black-colored bars) using canonical cell type-specific markers. (**d**) After cluster consolidation, a final annotation was provided for individual cells. (**e**) The average expression (+/− standard error) of each cluster is summarized. Gene-by-gene expression figures are available at http://betsholtzlab.org/VascularSingleCells/database.html. Figure c–d overlap with Extended Data Figure 1b–c,i in our related publication (ref. 4).

The blood vasculature is built from two principal cell classes: endothelial cells, which line the blood vessel lumens, and mural cells, which surround and/or stretch along the endothelial tubes. Mural cell is a collective term for pericytes and vascular smooth muscle cells (SMCs). Pericytes are broadly defined as the mural cells of microvessels, whereas SMCs occupy arteries and veins. In spite of clear differences in anatomical location and cell morphology, insight into the molecular and functional differences of mural cell subtypes is still limited^1,2^. Concerning other vessel-associated cell types, large arteries and veins harbor a clearly distinguishable outer layer–the adventitia–that contains fibroblast-like cells and extracellular matrix (ECM). However, a more exact definition of the adventitial ECM-producing cells, functionally as well as transcriptomically, is still missing. The presence of adventitial cells along smaller arterial and venous branches is also poorly understood. In the mouse brain, a new type of perivascular/leptomeningeal cell was recently pinpointed^3^, but a more exact anatomical and molecular description of these cells was still missing. In order to achieve a molecular understanding of the constituent cell types, using single cell RNA sequencing (scRNA-seq), we transcriptionally profiled vascular and vessel-associated cells in brain and lung^4^. Here, we provide a Data Descriptor for this dataset (Fig. 1a).

**Figure 2:**
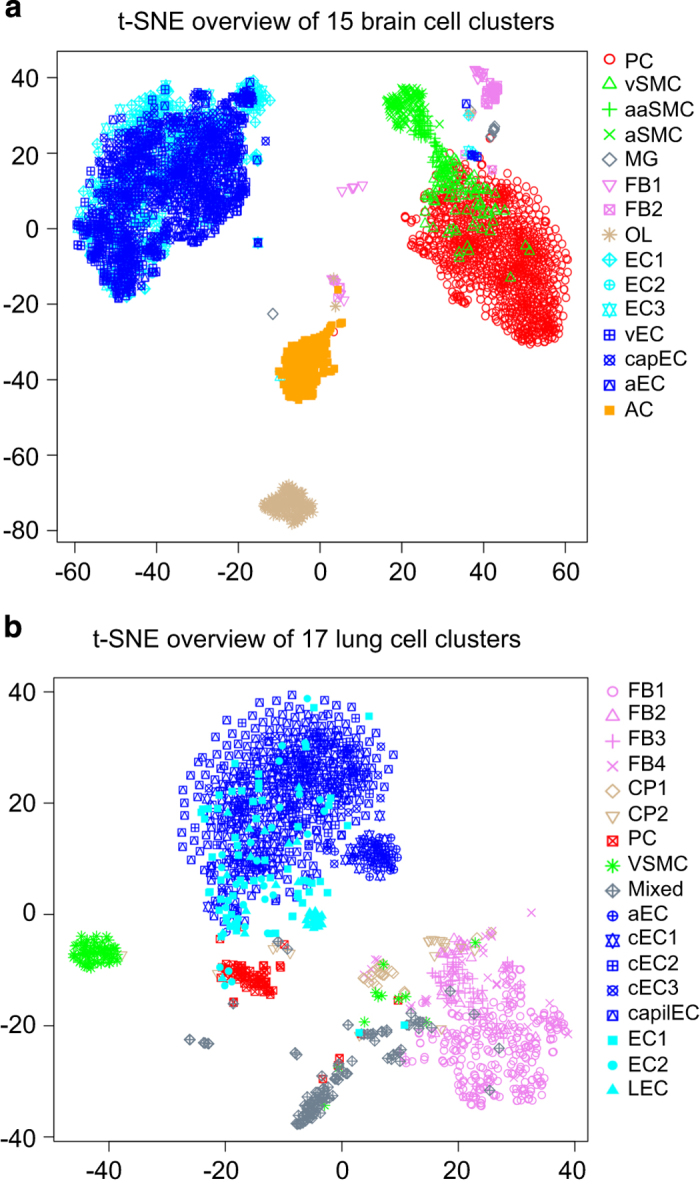
Overview of the single cell data in the adult mouse brain and lung. (**a**) The 3,418 brain single cells were analyzed by the T-Distributed Stochastic Neighbor Embedding (*t*-SNE) method to visualize their similarities, and the first two dimensions were used to plot the cells. Each cell is color-coded and also shape-coded according to its classified cell types from BackSPIN result annotation. (**b**) The same analysis of the 1,504 lung single cells as in panel **a**.

**Figure 3:**
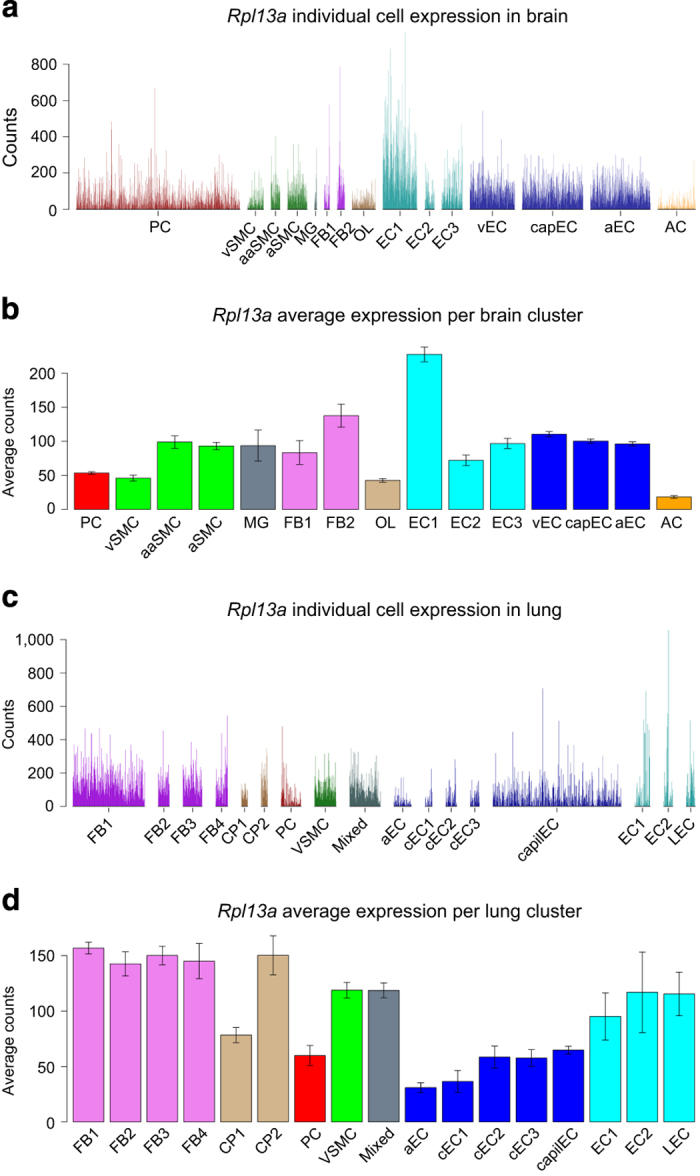
A screenshot of the database search outputs. An example search of ribosomal gene Rpl13a in the online database http://betsholtzlab.org/VascularSingleCells/database.html. Four figures are displayed. (**a**) The detailed expression in each cell in the brain dataset. (**b**) The average expression level in each of the 15 clusters in the brain. (**c**) The detailed expression in each cell in the lung dataset. (**d**) The average expression level in each of the 17 clusters in the lung.

To capture vascular and vessel-associated cell types from the adult mouse brain, we used a set of transgenic reporter mice: *Cldn5*(BAC)-GFP for endothelial cells, *Pdgfrb*(BAC)-eGFP;*Cspg4*-DsRed for mural cells and *Pdgfra*-H2BGFP for perivascular fibroblast-like cells (Fig. 1b). We also took advantage of an unexpected reporter gene expression in vessel-associated astrocytes from the *Tagln*-Cre;R26-stop-tdTomato mouse to capture vessel-associated astrocytes. To capture vascular and vessel-associated cell types from the adult mouse lung, we used *Cldn5*(BAC)-GFP for lung endothelial cells, *Pdgfrb*(BAC)-eGFP;*Cspg4-*DsRed and *Pdgfrb*(BAC)-eGFP for mural cells. Single fluorescent cells were sorted into 384-well plates, lysed, and the mRNA was converted into cDNA libraries using the SmartSeq2 protocol and sequenced^4^. We generated scRNA-seq transcriptomes from 3,436 single cells from the brain and 1,504 single cells from the lung (Data Citation 1). For each organ, the single cell transcriptomes were clustered using BackSPIN (Fig. 1c–e). After manual inspection and annotation, we defined 15 cell clusters in the brain, which following annotation using known canonical markers for the established vascular cells types along with validation of cell subtypes using immunofluorescence and *in situ* hybridization methods were found to correspond to: pericytes, three types of vascular smooth muscle cells (venous, arteriolar and arterial), microglia, two types of fibroblast-like cells, oligodendrocyte-lineage cells, six types of endothelial cells (venous, capillary, arterial and three others) and astrocytes (Fig. 2a). In the lung, we defined 17 cell clusters. Because our main objective with the lung dataset was to compare brain and lung pericytes, the annotation process of lung cells other than pericytes and endothelial cells was less extensive, but nevertheless indicated the existence of several subtypes of fibroblasts (split in four clusters) and cartilage/perichondrium-related cells (two clusters), pericytes (one cluster), vascular smooth muscle cells (one cluster), and at least two distinct types of endothelial cells (split into eight clusters) (Fig. 2b). To allow the scientific community to contribute to the further annotation of these cell types by assessing their gene expression, we provide user-friendly access to our data in the form of a searchable database http://betsholtzlab.org/VascularSingleCells/database.html, in which any gene can be searched by acronym, and its expression across the analyzed cell types in brain and lung displayed as single-cell bar-plots as well as diagrams displaying average values for the expression in the different cell types (see Fig. 3a-d for an example).

The dataset in this Data Descriptor provides a first comprehensive molecular profile of vascular and vascular-associated cell types in mouse brain, and a preliminary analysis of vascular and mesenchymal cell types in the lung, the latter complementing recently published single cell data on lung mesenchyme^5,6^. Our dataset provides a foundation for future studies of vascular development, homeostasis and diseases.

## Methods

The descriptions of the method protocols below are reproduced and extended from our related research publication^4^, with added details on computational data processing steps.

### Isolation of single cells

The preparation of the heart and lung tissue for single cell analysis has been described in our related publication^4^, as well as in the following three papers in Protocol Exchange: Brain cell isolation: DOI: 10.1038/protex.2017.159; Perivascular single cell isolation: 10.1038/protex.2018.005; Lung single cell isolation: 10.1038/protex.2018.006. In short, all tissues were disintegrated into single cell suspensions using a combination of enzymatic digestion and mechanical dissociation, followed by selection of the cells of interest using Fluorescence-Activated Cell Sorting (FACS; BD FACSAria III, BD Bioscience). Selected cells were deposited as single cells in 384-well plates, each well containing 2.3 μl of lysis buffer (0.2% Triton-X (Sigma, cat: T9284), 2U/μl RNase inhibitor (ClonTech, cat: 2313B), 2 mM dNTP’s (ThermoFisher Scientific, cat: R1122), 1 μM Smart-dT30VN (Sigma), ERCC 1:4 × 10^7^ dilution (Ambion, cat: 4456740)) prior to library preparation using the Smart-Seq2 protocol.

### Preparation of single cell sequencing libraries

An extended description of the Smart-Seq2 protocol can be found in Picelli *et al.*^7^. Briefly, mRNA was converted into cDNA through a reverse transcription (RT) reaction based on an oligo(dT) primer and the SuperScript II RT enzyme (ThermoFisher Scientific, cat: 18064-071). SuperScript II adds 2-5 untemplated cytosine nucleotides to the 3’ end, which enables the use of a Template-Switching Oligo (TSO) binding to the 3’ end of the first strand cDNA initiating the synthesis of full-length double-stranded cDNA. This cDNA was amplified with 22 cycles of PCR and the quality and quantity of the cDNA was assessed with a DNA high sensitivity chip on a BioAnalyser or TapeStation 4200 (Agilent Biotechnologies). When the sample plate passed the quality control, the cDNA was fragmented and tagged (i.e. tagmented) with the Tn5 transposase (Nextera XT library kit, Illumina, cat: FC-131-1096)^8^, and individual wells indexed using the Illumina Nextera XT indexing kits (Set A-D, Illumina, cat: FC-131-2001, FC-131-2002, FC-131-2003 and FC-131-2004). All libraries prepared for this study were sequenced on a HiSeq2500, using single 50 base pair reads and dual indexing.

### Alignment and generation of counts

The RNA-seq aligner, Spliced Transcripts Alignment to a Reference (STAR, version 2.4.2a) was used to align the short reads to the mouse reference genome (mm10)^9^. The aligner is available for downloading at https://github.com/alexdobin/STAR. Two-pass alignment was chosen to have improved performance of *de novo* splice junction reads, filtered for only uniquely mapping reads. The STAR parameters are as follows:

STAR --runThreadN 1 --genomeDir mm10 --readFilesIn XXX.fastq.gz --readFilesCommand zcat --outSAMstrandField intronMotif --twopassMode Basic

The expression values were computed per gene as described in Ramsköld et al.^10^, using uniquely aligned reads and correcting for the uniquely alignable positions using MULTo57(ref. 11). As QC threshold, cells with less than 100,000 reads were discarded, as well as cells that had a Spearman correlation below 0.3.

Our analyses and cell type annotations were based on 3,186 brain vascular-associated cells, 1,504 lung vascular-associated cells and 250 brain astrocytes, which were obtained in parallel experiments using different reporter mice and partly different procedures to obtain the cells (see ref. 4). Therefore, in order to compare the gene expression counts across different cells, the total gene counts for each cell were normalized to 500,000. The R code used for the normalization is available in the Supplementary File 1. The R tsne packages (version 0.1.3) was applied to visualize the 2D t-SNE map and GGally packages (version 1.3.1) was used to make gene pairs plot.

### Cell type classification with BackSPIN

As a clustering method, the BackSPIN algorithm^12^ was applied to classify the cells into different cell types. The BackSPIN software was downloaded from https://github.com/linnarsson-lab/BackSPIN (2015 version). BackSPIN was run with the following parameters:

### backspin -i input.CEF -o output.CEF -v -d 6 -g 3 -c 5

This iteratively splits the cells into six levels. After manual inspection and annotation, we defined 15 cell clusters in the brain and 17 cell clusters in the lung^4^.

### Online database construction

The expression database was constructed using html and javascript. For each gene, four figures were pre-made and stored on the server for faster display (see Fig. 3a-d for an example), including: the detailed expression in each cell in the brain dataset (Fig. 3a); the average expression level in each of the 15 clusters in the brain (Fig. 3b); the detailed expression in each cell in the lung dataset (Fig. 3c) and the average expression level in each of the 17 clusters in the lung (Fig. 3d). The gene symbol auto-complete function was implemented using the jquery.autocomplete.min.js and jquery-1.9.1.min.js plugin (available from https://github.com/devbridge/jQuery-Autocomplete/). The html page source and javascript code of the online database is available online at http://betsholtzlab.org/VascularSingleCells/database.html.

In order to identify enriched genes in specific brain cell type(s), the average expression for each cell types was stored in a MySQL (version 5.0.12-dev) database table and user queries were passed through a PHP (version 7.0.23) script to the MySQL database.

### Code availability

The R code used to process the sequencing data and visualize the results is available in the Supplementary File 1 (R version 3.3.2).

## Data Records

The information table for all the cells used in this study is available on Figshare (Data Citation 1). All sequence data and counts matrixes have been deposited in Gene Expression Omnibus database (Data Citation 2–3–4).

## Technical Validation

### Quality control of single cell sequencing cDNA and libraries

For each experiment, two different plate layouts were used for the FACS-based sorting. One plate (termed the ‘sample plate’) received one cell in each well of a 384 well plate and was used to obtain the data. The other plate (referred to as the ‘validation plate’) only contained lysis buffer in the first two columns, and received cells in the following pattern: Twenty cells in A1 and A2, no cells in P1 and P2, and one cell in the rest of column 1 and 2. The validation plate was used both as a quality control for sorting efficiency as well as allowing cDNA amplification optimization prior to proceeding with all 384 cells of the sample plate.

**Figure 4:**
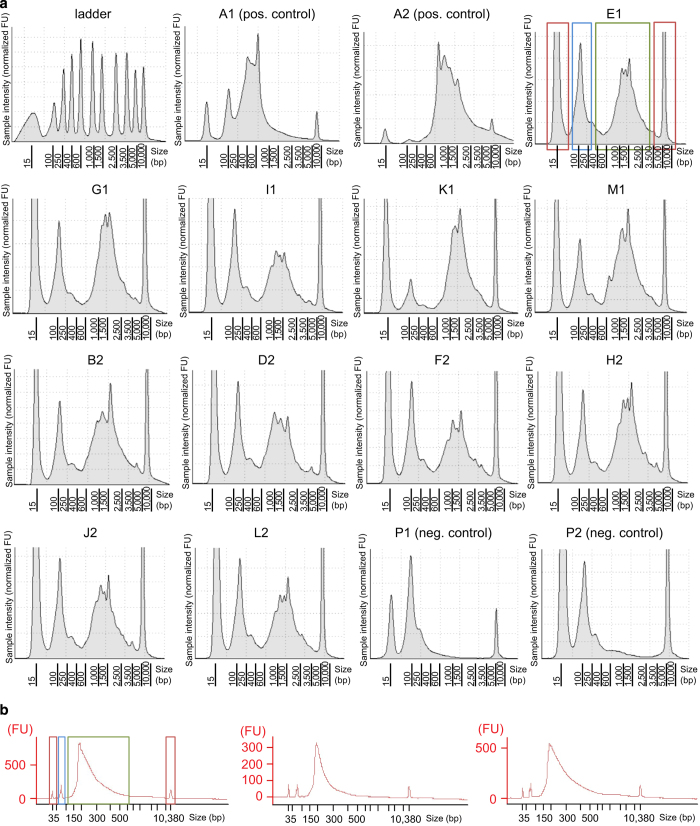
Quality control of the single cell sequencing library preparation. (**a**) Representative cDNA graphs from a validation plate, analyzed with a High Sensitivity D5000 ScreenTape on a TapeStation 4200. The size distribution of the cDNA was established by running a ladder (top left). A1 and A2 represent positive controls (20 cells), while P1 and P2 are empty negative controls. The other graphs display cDNA size distribution of randomly picked single cells of a validation plate after amplification. Graph E1 has colored boxes to assist in clarifying peak significance: Red boxes indicate upper and lower markers for size selection, the green box shows the cDNA size distribution and the blue box highlights the primer dimers. (**b**) Quality control of the pooled sequencing library after tagmentation with homemade Tn5 and indexing with the Nextera Indexing XT kit. The sequencing pools were analyzed on a BioAnalyzer with a High Sensitivity DNA Chip. Colored boxes describe peak significance as described above.

It is impossible to reliably measure mRNA quantity and quality of a single cell without prior amplification of the minute amount of RNA, and thus the first quality control check was done on the validation plate after cDNA synthesis and 22 PCR cycles of amplification. The cDNA quality and concentration were assessed using a DNA high sensitivity assay on a TapeStation 4200 or BioAnalyzer (Agilent Technologies) (Fig. 4a). A major size distribution around 1,500 base pairs indicated intact mRNAs and good quality of cDNA synthesis, sufficient for library preparation. A large cDNA size distribution between 100 and 500 base pairs indicated mRNA degradation and was not processed for library synthesis (data not shown). If the validation plate passed the quality control, the sample plate was processed in the same way as the validation plate. If needed, the PCR cycles could be increased to enrich the cDNA further, yet this has proven unnecessary in this study. After tagmentation and indexing of the cDNA, the libraries were pooled and assessed for quantity and quality with a High Sensitivity DNA chip on a BioAnalyzer (Agilent Biotechnologies) (Fig. 4b).

### Technical validation of the data

**Figure 5:**
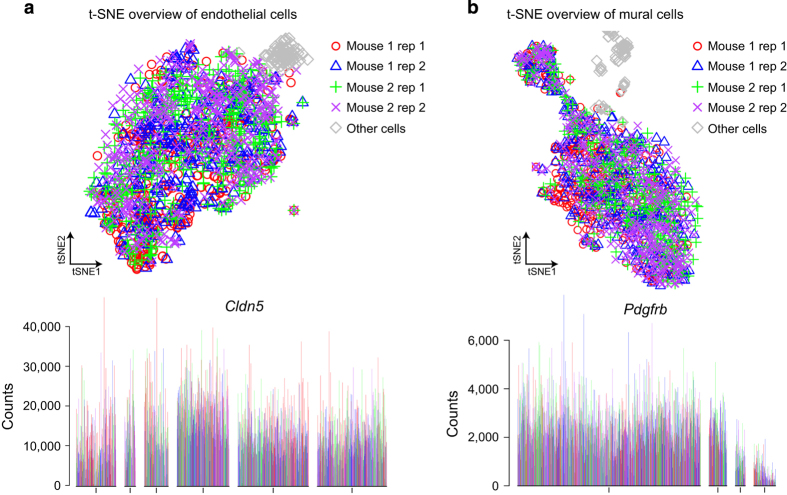
The distribution of replications of endothelial cells and mural cells. (**a**) The mouse origin and the technical plate replicates for the four plates of mural cells were color-coded in t-SNE and bar-plot display. The strong endothelial cell marker gene *Cldn5* is illustrated. (**b**) The same analysis of the four plates of mural cells as in **a**. The strong mural cell marker *Pdgfrb* is illustrated.

Beyond the quality control measures described above, additional steps were taken to ensure the validity of the data. To validate the clustering result based on BackSPIN result, the brain and lung single cell data were independently analyzed by the T-Distributed Stochastic Neighbor Embedding (*t*-SNE) method (Fig. 2a,b). In both the brain and lung data, the *t*-SNE result spread the single cells in 2-D space and revealed several cell groups. When overlaying and color-coding the cluster result from BackSPIN analysis on *t*-SNE result, the two methods showed good concordance in general. To check the possible batch effect for the major cell classes in the dataset (endothelial cells and mural cells), two sample plates were sorted per mouse, allowing assessment of technical variation between sample processing. As exemplified for the four plates of endothelial cells (Fig. 5a) and four plates of mural cells (Fig. 5b), the mouse origin as well as the technical replicates were color-coded. No significant differences could be found between the different plates or different animals when visualizing the data with *t*-SNE and displaying expression barplot of cells order by BackSPIN.

**Figure 6:**
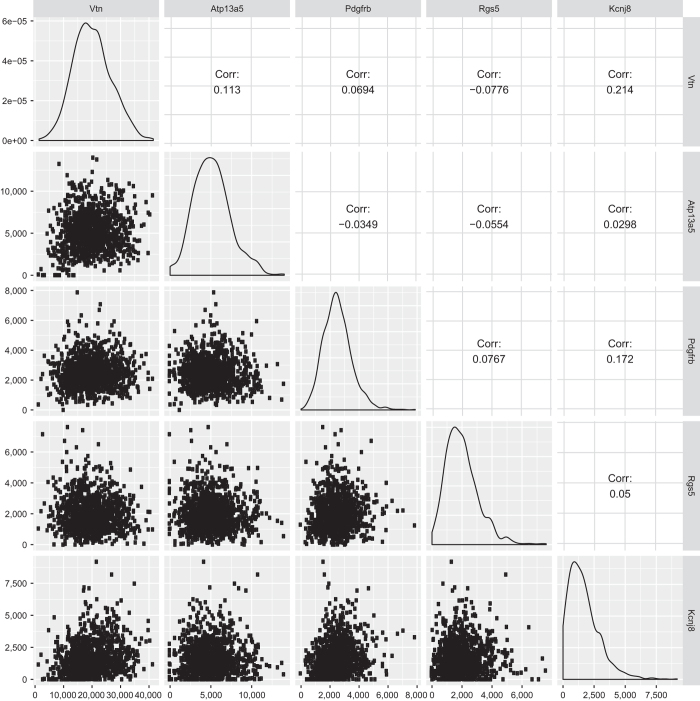
Analysis of the gene expression level of five pericyte-specific genes. The gene names are labeled on the top and right side of the figure. The five density plots in the diagonal line shows expression summary of each individual gene from all pericyte cells. The scatter plot on the lower left panels shows the pair-wise comparison of genes in each cell and the correlation coefficients are indicated on the upper right panels.

**Figure 7:**
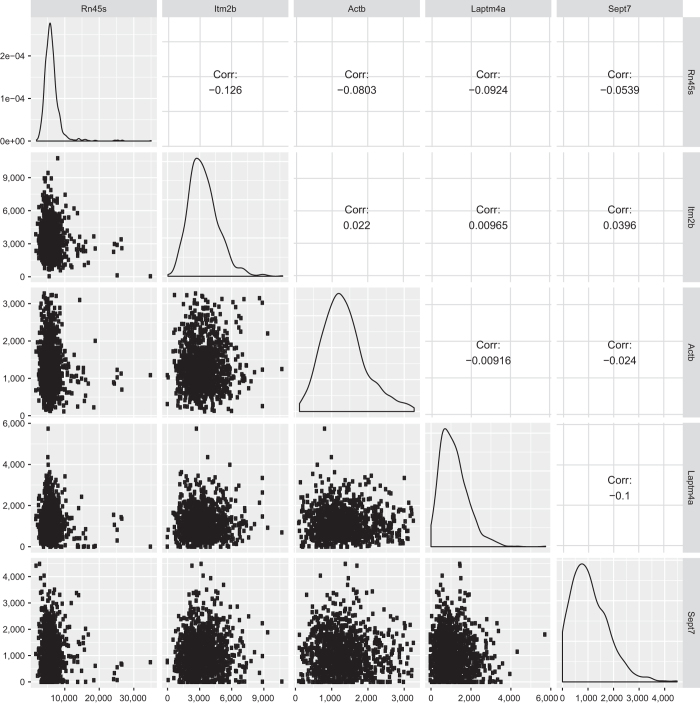
Analysis of the gene expression level of five broadly expressed genes. Description refers to Figure 6.

In our dataset, it is common to see a strong variation in gene expression levels between individual cells. These most likely reflect stochastic events from either biological origin (i.e. burst expression of genes) or experimental origin (incomplete capture rate of mRNA by Smart-Seq2 and strong PCR amplification). In order to rule out that inter-cellular differences in gene expression could be a reflection of library quality, we hypothesized that library quality would be correlated to gene expression. Therefore, we analyzed 5 highly expressed pericyte specific genes for correlation of expression of these genes within the cluster (Fig. 6). No correlation could be found, suggesting that inter-cellular differences of expression are not related to library quality. In addition, we also analyzed 5 genes that were broadly expressed in the whole dataset, and again, no correlation could be found (Fig. 7). Thus, we complement the QC on our dataset with a new type of analysis indicating that cell-to-cell variation of gene expression within the same cell population is a stochastic event.

## Additional information

**How to cite this article**: He, L. *et al*. Single-cell RNA sequencing of mouse brain and lung vascular and vessel-associated cell types. *Sci. Data* 5:180160 doi: 10.1038/sdata.2018.160 (2018).

**Publisher’s note**: Springer Nature remains neutral with regard to jurisdictional claims in published maps and institutional affiliations.

## Supplementary Material



Supplementary Information
