# Supplementary material for: Single-cell RNA sequencing of mouse brain and lung vascular and vessel-associated cell types
Source: Sci Data. 2018 Aug 21;5:180160. doi: 10.1038/sdata.2018.160 (PMC6103262; doi:10.1038/sdata.2018.160)
Supplement: Supplementary Information [file sdata2018160-s2.pdf]

Methods

Single cell raw counts normalization

From the single cell sequencing result, there were 3,186 brain vascular-associated cells, 1,504 lung vascular-associated cells, and 250 brain pure astrocytes obtained in a parallel study. In order to compare the gene expression counts across different cells, the total gene counts for each cell was normalized to 500,000.

The following R code (R version 3.3.2) was used for the counts normalization (eg: brain 3,186 cells):

```
brain.cell.sum=apply(Brain_samples_raw_read_counts_matrix, 2, sum)
scale.500K=as.numeric(brain.cell.sum)/500000
Brain_samples_normalized_counts_matrix
=t(t(Brain_samples_raw_read_counts_matrix) / scale.500K)
Brain_samples_normalized_counts_matrix
=round(Brain_samples_normalized_counts_matrix, 0)
```

The raw counts and normalized counts are available for downloading from Data Citation 2-4.

BackSPIN

The BackSPIN software was downloaded from:
https://github.com/linnarsson-lab/BackSPIN (2015)

Prepare the input counts data (e.g. Brain_samples_normalized_counts_matrix) into CEF format for BackSPIN using the following R code:

```
cef.dat= Brain_samples_normalized_counts_matrix
output.cef="brain_data.cef"
cef=cef.dat
cef=rbind(gene="", data.frame(well="", cef) )
cef.head=c("CEF",  "0", "1",
          "1", nrow(cef.dat), ncol(cef.dat), "0")
write.table(matrix(cef.head, nrow=1), output.cef, sep="\t", row.names=F,
col.names=F,quote=F)
write.table(cef, output.cef, sep="\t", row.names=T, col.names=NA, quote=F, append
= T)
rm(cef.dat)
rm(cef)
```

BackSPIN was run with the following parameters: -d 6 -g 3 -c 5, splitting the cells into six levels. The cluster output CEF file for brain data is "brain_data_6Level_clustered.cef"

Clustering result visualization

To visualize backSPIN clustering result for both genes and cells, the output result was imported into R for data processing. The following R code was used:

```
cef=read.csv("brain_data_6Level_clustered.cef", header=F, sep="\t")
cef=as.matrix(cef)

cef.dat=cef[11:nrow(cef), 10:ncol(cef)]
cef.dat=t(apply(cef.dat, 1, as.numeric))
colnames(cef.dat)=as.vector(cef[2, 10:ncol(cef)])
rownames(cef.dat)=as.vector(cef[11:nrow(cef), 1])

cef.gene.k=cef[11:nrow(cef), 3:8]
cef.gene.k=t(apply(cef.gene.k, 1, as.numeric))
rownames(cef.gene.k)=as.vector(cef[11:nrow(cef), 1])
colnames(cef.gene.k)=1:6

cef.cell.k=t(cef[4:9, 10:ncol(cef)])
cef.cell.k=t(apply(cef.cell.k, 1, as.numeric))
rownames(cef.cell.k)=as.vector(cef[2, 10:ncol(cef)])
colnames(cef.cell.k)=paste("CellType_L",1:6, sep="")
```

The ordered count matrix was stored in variable 'cef.dat', and genes and cells clustering result were stored in 'cef.gene.k' and 'cef.cell.k', respectively.

BackSPIN dichotomously splits the cells. The R graph (version 1.52.0) and Rgraphviz (version 2.18.0) packages were utilized to visualize the hierarchical tree structures of the splitting process (Fig. 1c).

```
library(graph);
library(Rgraphviz)

cef.cluster.uniq=unique(cef.cell.k )
cef.cluster.uniq=cbind(0, cef.cluster.uniq)

net.info=cef.cluster.uniq
for(i in 1:ncol(cef.cluster.uniq)){
  net.info[,i]=paste(paste("L", i-1, sep=""), net.info[, i], sep=": ")
}

myNodes = unique(as.vector(net.info))

g <- new("graphNEL", nodes =myNodes, edgemode = "directed")
for(i in 1:(ncol(net.info)-1) ) {
  t.up=unique(as.vector(net.info[, i]));
  for(j in 1:length(t.up)){
    t.sub=unique(as.vector(net.info[net.info[,i]==t.up[j], i+1]))
    if(length(t.sub)>=1){
      for(s in t.sub){ g <- addEdge(t.up[j], s, g, 1) }
```

```
    }
  }
}

plot(g)
```

The gene expression profile in different clusters was visualized with bar plot using the following R code. Every bar represents the expression level in a single cell at a fixed position on the x-axis (Fig. 1c).

```
nk=length(unique(cef.cell.k[, "CellType_L6"]))
brain.spin.nk.n= as.numeric(unlist(lapply(0:(nk-1),
function(x){sum(cef.cell.k[,6]==x)})) )
t.space=50
n.c=ncol(cef.dat)
brain.spin.nk.space=c(0, unlist(lapply(brain.spin.nk.n, function(x){c(rep(0, x-1),
t.space)})))
brain.spin.nk.space=rev(rev(brain.spin.nk.space)[-1])
brain.spin.nk.lab.p=cumsum(brain.spin.nk.n)-(brain.spin.nk.n/2)

#to make barplot of an example gene "Actb"
t.g="Actb"
barplot(as.numeric(cef.dat[t.g,]), ylab = "counts",space=brain.spin.nk.space,
col="black",main=t.g,las=2, border = NA)
barplot(rep(0, n.c), col="grey",  space=brain.spin.nk.space,add=TRUE, axes=F)

for(p in 1:nk){
   axis(1, brain.spin.nk.lab.p[p]+t.space*(p-1), paste("", p-1, sep=""), cex.axis=0.8)
}
```

For the brain cells, based on the above BackSPIN result of six level split, after manual inspection, we further splitted heterogeneous clusters using BackSPIN again, and consolidated small or hyper-split clusters to a higher node in the tree. Also, we added 250 pure brain astrocyte cells that were obtained in a parallel study. In the end, there were 15 different brain clusters in total. The following R code was used to visualize the expression level in each of the brain single cell at a fixed position on the x-axis (Fig. 1d):

```
# to assign the cells into 15 cell types.
my.cell.15groups=rbind(
  data.frame(type="PC", cells=PC.cell.ids.from.sub.clustering),
  data.frame(type="vSMC", cells= vSMC.cell.ids.from.sub.clustering),
  data.frame(type="aaSMC", cells=rownames(cef.cell.k[cef.cell.k[,6] %in% 5:6,]) ),
  data.frame(type="aSMC", cells= rownames(cef.cell.k[cef.cell.k[,6]==7,]) ),
  data.frame(type="MG", cells=rownames(cef.cell.k[cef.cell.k[,6]==2,]) ),
  data.frame(type="FB1", cells=rownames(cef.cell.k[cef.cell.k[,6]==3,])  ),
```

```
  data.frame(type="FB2", cells=rownames(cef.cell.k[cef.cell.k[,6]==4,])  ),
  data.frame(type="OL", cells=rownames(cef.cell.k[cef.cell.k[,6] %in% 8:10,]) ),
  data.frame(type="EC1", cells= rownames(cef.cell.k[cef.cell.k[,6] %in% 11:16,]) ),
  data.frame(type="EC2", cells= rownames(cef.cell.k[cef.cell.k[,6] %in% 17:20,]) ),
  data.frame(type="EC3", cells= rownames(cef.cell.k[cef.cell.k[,6] %in% 21:24,]) ),
  data.frame(type="vEC", cells= rownames(cef.cell.k[cef.cell.k[,6] %in% 25:28,]) ),
  data.frame(type="capilEC", cells= rownames(cef.cell.k[cef.cell.k[,6] %in% 29,]) ),
  data.frame(type="aEC", cells= rownames(cef.cell.k[cef.cell.k[,6] %in% 30:37,]) ),
  data.frame(type="AC", cells= astrocyte.250cell.ids )
  )

# set the bar color for each of the 15 cell types.
my.cell.col.15groups=rbind(
  data.frame(type="PC", cols="darkred"),
  data.frame(type="vSMC", cols="darkgreen"),
  data.frame(type="aaSMC", cols="darkgreen"),
  data.frame(type="aSMC", cols="darkgreen"),
  data.frame(type="MG", cols="darkslategrey"),
  data.frame(type="FB1", cols="darkviolet"),
  data.frame(type="FB2", cols="darkviolet"),
  data.frame(type="OL", cols="tan4"),
  data.frame(type="EC1", cols="darkcyan" ),
  data.frame(type="EC2", cols="darkcyan" ),
  data.frame(type="EC3", cols= "darkcyan"),
  data.frame(type="vEC", cols="darkblue"),
  data.frame(type="capilEC", cols= "darkblue" ),
  data.frame(type="aEC", cols= "darkblue"),
  data.frame(type="AC", cols="darkorange")
)

my.order=as.vector(unique(my.cell.15groups[,1]))
my.cell.order=as.vector(my.cell.15groups[,2])
nk=length(my.order)

adj.spin.nk.n= as.numeric(unlist(lapply(my.order,
function(x){sum(my.cell.15groups[,1]==x)})) )
t.space=50
n.c=ncol(brain.db.dat)

adj.spin.nk.space=c(0, unlist(lapply(adj.spin.nk.n, function(x){c(rep(0, x-1),
t.space)})))
adj.spin.nk.space=rev(rev(adj.spin.nk.space)[-1])
adj.spin.nk.lab.p=cumsum(adj.spin.nk.n)-(adj.spin.nk.n/2)

#plot an example of Actb gene
t.g="Actb"
```

```
barplot(as.numeric(brain.db.dat[t.g, my.cell.order]), space=adj.spin.nk.space,
col="black", main=t.g,border = NA, las=2)
barplot(rep(0,n.c), col="grey",  space=adj.spin.nk.space,add=TRUE, axes=F)
#add label to each cluster
for(p in 1:nk){
  axis(1, adj.spin.nk.lab.p[p]+t.space*(p-1), paste(my.order[p],
adj.spin.nk.n[p],sep="\n"), cex.axis=0.6)
}
```

In order to better visualize expression differences between the 15 brain cell types or clusters, gene bar plots were created using the average read count from all cells within each group (Fig. 1e). The following R code was used:

```
# average expression in each cluster
L.clusters =  my.order

g.all.k.mean= do.call(cbind,
                      lapply(L.clusters, function(i){
                        cell.k=as.vector(my.cell.15groups[my.cell.15groups[,1]==i, "cells"]);
                        gene.cell.k.mean=apply(brain.db.dat[, cell.k], 1, mean)
                      }))
colnames(g.all.k.mean)=L.clusters

std <- function(x) sd(x)/sqrt(length(x))

g.all.k.mean.std= do.call(cbind,
                          lapply(L.clusters, function(i){
                            cell.k=as.vector(my.cell.15groups[my.cell.15groups[,1]==i,
"cells"]);
                            gene.cell.k.std=apply(brain.db.dat[, cell.k], 1, std)
                          }))
colnames(g.all.k.mean.std)=L.clusters

my.cell.col.15groups.bar=rbind(
  data.frame(type="PC", cols="red"),
  data.frame(type="vSMC", cols="green"),
  data.frame(type="aaSMC", cols="green"),
  data.frame(type="aSMC", cols="green"),
  data.frame(type="MG", cols="slategrey"),
  data.frame(type="FB1", cols="violet"),
  data.frame(type="FB2", cols="violet"),
  data.frame(type="OL", cols="tan"),
  data.frame(type="EC1", cols="cyan" ),
  data.frame(type="EC2", cols="cyan" ),
  data.frame(type="EC3", cols= "cyan"),
  data.frame(type="vEC", cols="blue"),
  data.frame(type="capilEC", cols= "blue" ),
  data.frame(type="aEC", cols= "blue"),
```

```
  data.frame(type="AC", cols="orange")
)

library(gplots)

t.g="Actb";

barplot2( as.numeric(g.all.k.mean[t.g,]), plot.ci = TRUE,
          ci.l =  as.numeric(g.all.k.mean[t.g,])- as.numeric(g.all.k.mean.std[t.g,]),
          ci.u =  as.numeric(g.all.k.mean[t.g,])+ as.numeric(g.all.k.mean.std[t.g,]),
          ci.width=0.2, main=g.title,
          col=as.vector(my.cell.col.15groups.bar$cols),names.arg=my.order,las=1,
cex.names=0.8,  xaxs = "i",  xlim = c(0, 18), ylab="Average counts"
  )
```
